# Maternal Mortality: 10 Year Experience of a Tertiary Center in Turkey

**DOI:** 10.1155/2020/3595024

**Published:** 2020-09-30

**Authors:** Erdem Fadiloglu, Canan Unal, Atakan Tanacan, Serpil Ocal, Banu Kilicaslan, Seda Banu Akinci, Arzu Topeli, M. Sinan Beksac

**Affiliations:** ^1^Division of Perinatology, Department of Obstetrics and Gynecology, Hacettepe University Medical Faculty, Ankara, Turkey; ^2^Division of Critical Care, Department of Internal Medicine, Hacettepe University Medical Faculty, Ankara, Turkey; ^3^Division of Critical Care, Department of Anaesthesiology and Reanimation, Hacettepe University Medical Faculty, Ankara, Turkey

## Abstract

We retrospectively evaluated five maternal mortality cases that occurred in our institution within the last 10 years. Rate of maternal mortality was 24.5 per 100000 live births. Maternal mortality causes were cardiopulmonary failure secondary to veno-occlusive disease, septic shock secondary to osteosarcoma, pulmonary thromboembolism secondary to metastatic breast cancer, septic shock secondary to cholecystitis, and postpartum hemorrhage secondary to Niemann–Pick disease. Four out of five cases were evaluated as indirect maternal mortality cases. Three out of five cases ended up with a healthy newborn, while other cases ended up with abortus and postpartum exitus.

## 1. Introduction

Preventing maternal mortality and providing optimal outcomes both for mother and fetus are the main concerns of all obstetricians. Health policies and clinical management protocols are being updated for these purposes. Maternal mortality rates are also evaluated as one of the most important parameters showing the quality of health services in countries or regions. However, maternal mortality is still a critical health issue in both low- and middle-income countries [1]. According to recent data, maternal mortality rate has been determined as 13.7 in 2015 and 14.6 in 2017 per 100.000 live births, respectively, in Turkey [2,3].

Some certain risk factors such as advanced maternal age or coexistent systemic disorders like diabetes mellitus, hypertension, or cardiovascular diseases are reported to be associated with maternal mortality in previous literature [4]. In this paper, we have presented the maternal mortality cases of our institution in between 2009 and 2019. We believe that maternal mortality rates of national institutions must be known in order to have better health care planning.

This study was approved by Ethical Committee of our institution with the approval number of GO: 19-835.

## 2. Case Reports

We have retrospectively evaluated the medical records of five maternal mortality cases that took place at Hacettepe University, Ankara, Turkey. Our institution is working as a referral center for both fetal and maternal health problems and giving health care for patients all over the country with multidisciplinary approach with availability of intensive care unit (ICU) and neonatal intensive care unit (NICU). Rate of maternal mortality was 24.4 per 100000 live births for this time interval in our institution.  Table 1 presents a summary of the cases and gives further details of each case.

### 2.1. Case 1

An eighteen-year-old patient was admitted to the emergency room for dyspnea at the 29th gestational week during her first pregnancy. The patient had been hospitalized one week before at another institution with a diagnosis of pulmonary thromboembolism (PTE) and had been using low-molecular-weight heparin (LMWH) at a dose of 0.6 mL twice a day and had been referred to our institution due to newly onset generalized edema and increased liver function tests. Her initial evaluation revealed tachypnea and tachycardia with oxygen saturation of 92% with a nasal cannula (2 L/minute) at arterial blood gas analysis and impaired liver function tests. Physical examination revealed petechiae, ecchymosis, and generalized edema at whole body. Prediagnosis was determined as PTE at the emergency room, and the patient got hospitalized to the intensive care unit (ICU). Inotropic agents were administered on the first day of hospitalization and intubated due to persistent low-oxygen saturations despite oxygen support. The patient had resistant hypotension during invasive mechanical ventilation and performed computerized thoracic tomography created suspicion for the veno-occlusive disease instead of PTE. Echocardiography revealed a right-sided heart failure, and systolic pulmonary artery pressure was 105 mmHg. Despite supportive treatments and invasive mechanical ventilation, blood gas pH declined to 6.9 and cardiopulmonary resuscitation (CPR) has been performed due to cardiac arrest. Emergency cesarean section has been performed at ICU after the acquirement of informed consent from the family, and a 1570 g male infant has been delivered with an APGAR score of 1 and cyanosis and has been accepted as exitus after resuscitation. The patient was also accepted as exitus after one hour of resuscitation at her first day of admission. An autopsy was not performed due to the lack of consent from the family.

### 2.2. Case 2

A thirty-three-year-old nulliparous patient was hospitalized at the 28th gestational week due to lethargy. The patient had a history of osteosarcoma and a leg amputation with lung metastasis resulting in 4 surgeries and multiple chemotherapy sessions which was initially diagnosed and operated 12 years ago. At the time of admission, the patient had pancreatic metastasis and epigastric mass protruding stomach anteriorly. Initial laboratory analysis revealed a serum creatinine of 9.67 mg/dL and a hemoglobin level of 7.9 g/dL. Obstetric ultrasonography revealed singleton pregnancy with normal findings. The patient had been hospitalized to ICU due to severe dyspnea on the 3rd day of hospitalization. Regular contractions had begun at this period, and the patient delivered by vaginal route at ICU. Newborn had APGAR scores of 7 and 8 at the first and fifth minutes, respectively, and birth weight of 1200 g. The patient was followed up at ICU at the postpartum period for respiratory and renal functions and required multiple sessions of hemodialysis at this period. However, renal functions and respiratory problems worsened at the forthcoming period, and this patient got intubated at the 13th day of hospitalization. Despite these efforts, the patient died 2 days after intubation due to sepsis. The newborn was discharged from NICU after hospitalization for 29 days due to prematurity.

### 2.3. Case 3

A twenty-nine-year-old patient was referred to our clinic with newly diagnosed breast cancer with liver metastasis at the 32nd gestational week. Initial evaluation of the patient revealed moderately increased liver function tests, and she had hypoxemia during hospitalization and was evaluated as PTE at the 33rd gestational week despite ongoing LMWH treatment. The emergency cesarean section was performed, and she was transferred to ICU. LMWH doses were revised as 0.6 ml twice a day. However, the patient got resistant hypotension and hypoxemia at this period, and the patient died despite of CPR at ICU. The newborn was discharged from NICU after 10 days of hospitalization.

### 2.4. Case 4

A twenty-five-year-old patient was referred to our institution with ongoing fever and abdominal pain at the 16th gestational week. The primary evaluation revealed anemia, and ultrasonographic imaging showed increased wall thickness and polyp at gall bladder. An emergency laparoscopic cholecystectomy was performed, but she was admitted to ICU due to tachycardia (103/minute) and tachypnea (40/minute) after the surgery with the initial diagnosis of sepsis. Chest X-ray showed adult respiratory distress syndrome findings, and the patient was intubated. Despite supportive treatment including i.v fluids, vasopressor agents, and invasive mechanical ventilation, the patient had a cardiac arrest that returned to normal cardiac rhythm after 2 minutes of CPR at the postoperative 3rd day. The patient got a spontaneous abortion at this period. Resistant vaginal bleeding persisted despite the lack of any placental retention. Due to concurrent radial artery occlusion and necrosis at distal phalanxes, intravenous heparinization had been performed with intermittent erythrocyte transfusion. However, intravenous heparin had been stopped due to sudden onset hemorrhage from larynx. Further red blood cells and fresh frozen plasma transfusions had been performed with supplementary intravenous hydration therapy. She had been followed up for 38 days at ICU with supportive treatments, and an amputation surgery had been planned for radial artery occlusion, but the patient had died due to cardiac arrest prior to surgery.

### 2.5. Case 5

A twenty-three-year-old nulliparous patient was referred to our hospital at the postpartum period. She had been performed an emergency cesarean section due to obstructed labour. A healthy newborn with APGAR scores (first and fifth minutes) of 9 and 9 was delivered. Tachycardia (130/minute) and severe anemia (Hb: 4 g/dL) had been detected at the postpartum period, and emergent hysterectomy was performed. The patient had cardiac arrest during the surgery due to hemodynamic shock and was referred to ICU in our institution. The patient could not be extubated after the surgery, and neurological examination revealed early signs of cerebral ischemia. Glasgow Coma Scale was evaluated as 3, and mechanic ventilation had been applied. The patient was diagnosed as multiple organ failure due to hypovolemic shock. Fluid replacement and positive inotropes were used for hemodynamic stability. During this hospitalization, studies regarding the etiology of postpartum hemorrhage revealed a history of splenectomy at the age of 16 and further studies unveiled the diagnosis of Niemann–Pick disease type B. On the 14th day of ICU hospitalization, brain death was confirmed, and the patient died 2 days after [5].

## 3. Discussion

It has been reported that maternal mortality rate has been determined as 13.7 and 14.6 per 100.000 live births in 2015 and 2017, respectively, in Turkey [2,3]. Postpartum hemorrhage and maternal cardiovascular diseases were found to be most frequent reasons for maternal mortality in Turkey [6]. Obstetrical complications are still the main reasons of maternal mortality in Turkey, while maternal health disorders are becoming more and more important in countries with better health organisations [7]. The maternal mortality rate in our institution is slightly higher than our national rates. This is most probably due to our institutional characteristics as our institution works as a tertiary referral center and gets referrals from all over the country both at the antenatal and postpartum periods.

In this report, we have presented five mortality cases that took place at our institution during the last decade. These cases were all associated with additional health problems with rare diseases resulting in maternal mortality beyond obstetric complications. Case 1 resulted in mortality due to cardiopulmonary failure secondary to veno-occlusive disease. Previous literature demonstrated that veno-occlusive disease may aggravate during the pregnancy as the pregnancy itself is a hypercoagulable state [8]. Excessive antithrombotic therapies are recommended at these patients for the prevention of venous coagulopathies. However, in our case, the diagnosis was made during pregnancy and resulted in mortality.

Cases 2 and 3 were patients with metastatic malignancies and resulted in pulmonary thromboembolism and septic shock. Pregnant patients with malignancy must be evaluated carefully as the maternal mortality rate may be as high as 31% [9]. Preconceptional counseling and providing remission or cure may be life-saving in these patients by avoiding mortality due to the disease itself or complications secondary to malignancy. Avoiding a pregnancy at active stage of the disease may be life-saving both for mother and fetus in certain circumstances. The necessity of chemotherapy and/or surgery must also be considered and planned carefully in such cases [10]. The timing of curative surgery must also be planned cautiously to have better outcomes in necessary cases. We have previously demonstrated that breast and thyroid cancers are the most frequently observed malignancies during pregnancy [11].

Case 4 is also indirect maternal mortality due to septic shock secondary to emergent cholecystectomy operation. Previous literature demonstrated similar rates of postoperative infection or sepsis after laparoscopic operations between pregnant and nonpregnant patients [12]. However, this case was complicated with adult respiratory distress syndrome which has a mortality rate of over 40% in pregnancy [13].

Case 5 resulted in maternal mortality due to resistant postpartum hemorrhage secondary due to Niemann–Pick disease [5]. Postoperative bleeding was reported in many cases for Niemann–Pick patients as a result of bone marrow implantation of the disease, thrombocytopenia, or bleeding disorders [14].

In our series, four out of five cases resulted in mortality due to indirect causes, while only one case resulted in maternal mortality due to a direct cause which was postpartum hemorrhage. However, we have also detected an indirect underlying disease causing the postpartum hemorrhage. Indirect causes of maternal mortality are reported to be common in developed countries such as the United Kingdom [15]. Contribution of indirect causes to maternal mortality also seems to be increasing in USA in recent years [16]. Establishing proper health policies and patient referral systems are important factors for declining maternal mortality [17]. The etiological factors behind mortality rates in developed countries indicate that obstetrical complication-related maternal mortality cases might be prevented by well-organized antenatal care programs. Prenatal counseling may also decrease the mortality rates in patients with systematic diseases.

Antenatal care is critical in terms of preventing maternal mortality from indirect causes. It has also been reported that the absence of antenatal care is significantly related to an increased rate of maternal mortality [1]. Our experience may show the importance of the multidisciplinary approach and interinstitutional network at high-risk pregnancies as all of our cases had an underlying disease resulting in maternal mortality. Early referral of these patients to tertiary centers is also critical for better planning.

Data related to neonatal outcomes in maternal mortality cases are limited in previous literature. Three out of five cases ended up with healthy newborns in our series, while other cases resulted in abortion or postpartum neonatal death. According to our results, we may suggest that neonatal outcome is strictly related to a gestational week at delivery and the cause of maternal mortality.

In conclusion, indirect maternal mortality causes still seem to be challenging for physicians, and near-miss events must be considered by a multidisciplinary approach for preventing maternal mortality. Antenatal care is also critical for family planning and preventing mortalities due to indirect causes.

## Figures and Tables

**Table 1:** Clinical characteristics and neonatal outcomes of the patients.

	Age	Gravida	Parity	Indications for hospitalization	Causes of mortality	Length of stay in hospital/day	Gestational age at delivery	Neonatal outcome
Case 1	18	1	0	Pulmonary veno-occlusive disease	Cardiopulmonary failure	1	29	Exitus
Case 2	33	1	0	Metastatic osteosarcoma	Septic shock	15	30	Delivery-discharged from NICU
Case 3	29	1	0	Metastatic breast cancer	Pulmonary thromboembolism	10	32	Delivery-discharged from NICU
Case 4	25	2	1	Cholecystitis	Septic shock	38	16	Abortus
Case 5	23	1	0	Postpartum hemorrhage	Niemann–Pick, hemorrhagic shock	16	40	Delivery-healthy newborn
